# Genome-wide SNPs reveal novel patterns of spatial genetic structure in *Aedes albopictus* (Diptera Culicidae) population in China

**DOI:** 10.3389/fpubh.2022.1028026

**Published:** 2022-11-10

**Authors:** Yong Wei, Song He, Jiatian Wang, Peiyang Fan, Yulan He, Ke Hu, Yulan Chen, Guofa Zhou, Daibin Zhong, Xueli Zheng

**Affiliations:** ^1^Department of Pathogen Biology, School of Public Health, Southern Medical University, Guangzhou, China; ^2^Clinical Laboratory, Shenzhen Qianhai Shekou Free Trade Zone Hospital, Shenzhen, China; ^3^Program in Public Health, College of Health Sciences, University of California, Irvine, Irvine, CA, United States

**Keywords:** invasive species, restriction site-associated DNA sequencing (RAD-Seq), genetic diversity, gene pool, isolation by distance

## Abstract

**Introduction:**

Since the second half of the 20th century, *Aedes albopictus*, a vector for more than 20 arboviruses, has spread worldwide. *Aedes albopictus* is the main vector of infectious diseases transmitted by *Aedes* mosquitoes in China, and it has caused concerns regarding public health. A comprehensive understanding of the spatial genetic structure of this vector species at a genomic level is essential for effective vector control and the prevention of vector-borne diseases.

**Methods:**

During 2016–2018, adult female *Ae. albopictus* mosquitoes were collected from eight different geographical locations across China. Restriction site-associated DNA sequencing (RAD-seq) was used for high-throughput identification of single nucleotide polymorphisms (SNPs) and genotyping of the *Ae. albopictus* population. The spatial genetic structure was analyzed and compared to those exhibited by mitochondrial cytochrome c oxidase subunit 1 (*cox*1) and microsatellites in the *Ae. albopictus* population.

**Results:**

A total of 9,103 genome-wide SNP loci in 101 specimens and 32 haplotypes of *cox*1 in 231 specimens were identified in the samples from eight locations in China. Principal component analysis revealed that samples from Lingshui and Zhanjiang were more genetically different than those from the other locations. The SNPs provided a better resolution and stronger signals for novel spatial population genetic structures than those from the *cox*1 data and a set of previously genotyped microsatellites. The fixation indexes from the SNP dataset showed shallow but significant genetic differentiation in the population. The Mantel test indicated a positive correlation between genetic distance and geographical distance. However, the asymmetric gene flow was detected among the populations, and it was higher from south to north and west to east than in the opposite directions.

**Conclusions:**

The genome-wide SNPs revealed seven gene pools and fine spatial genetic structure of the *Ae. albopictus* population in China. The RAD-seq approach has great potential to increase our understanding of the spatial dynamics of population spread and establishment, which will help us to design new strategies for controlling vectors and mosquito-borne diseases.

## Introduction

*Aedes (Stegomyia) albopictus* (Skuse, 1894), one of the 100 most dangerous invasive species worldwide, can be found on every continent except for Antarctica, because of global warming, human-aided transport, and insecticide resistance (1–3). The global spread of *Ae. albopictus* has attracted public health concern for mosquito-borne diseases. *Aedes albopictus* is a vector for more than 20 arboviruses, some of which are highly pathogenic and transmissible, for example, dengue virus, Chikungunya virus (CHIKV), and yellow fever virus (4).

*Aedes albopictus* as well as *Ae. aegypti* are responsible for the recent re-emergence of dengue and Chikungunya and new outbreaks of Zika virus infection (5, 6). *Aedes albopictus* is the predominant species found in nearly one-third of China (7, 8), whereas the distribution of *Ae. aegypti* is limited to small areas of southern China, including Hainan, Guangdong, Guangxi, and Yunnan Provinces (9–11). *Aedes albopictus* is the main vector of infectious diseases transmitted by *Aedes* mosquitoes in China. The threat of infectious disease transmission has led to the establishment of surveillance and vector control strategies, which gain from knowledge of the genetic, ecological, and behavioral traits of the mosquito populations (12).

Many studies on the genetic diversity and population structure of *Aedes albopictus* population have been conducted by using molecular markers such as mitochondrial gene cytochrome c oxidase subunit 1 (*cox*1) (13–20) and microsatellite markers (14, 21–23). However, the use of mitochondrial DNA as a marker in population, phylogeographic, and phylogenetic studies may be a problem due to the existence of inherited symbionts in *Ae. albopictus* (5, 24, 25). Microsatellite markers are codominant nuclear loci that are commonly used to infer levels of genetic diversity and population genetic structure in natural populations. However, bias selection for only the most polymorphic markers in the genome may result in reduced sensitivity and an inaccurate reflection of the underlying genome-wide levels of genetic diversity (26). Many population genetic studies have used mitochondrial DNA and microsatellite markers and provided limited resolution of population genetic structure patterns because of low levels of sequence variation or limited number of markers (5). For example, Gao et al. (14) and Wei et al. (27) reported two patterns of broad population structure on the basis of microsatellite markers alone and three major haplotype clusters of *cox*1 in the *Ae. albopictus* population in China. Genetic indices and environmental factors were combined and 17 *Ae. albopictus* populations across China were sampled and clustered into three groups that approximately correlated to three climate regions: tropical, subtropical, and temperature regions (14).

Currently, single nucleotide polymorphisms (SNPs), identified using next-generation sequencing techniques, are the markers of choice because of their abundance in the genomes of virtually all populations (28). Restriction site-associated DNA sequencing (RAD-seq) is one of the most popular strategies used to identify large numbers of bi-allelic SNPs in the genomes of vector mosquito populations. Several studies have been conducted using genome-wide SNPs to decipher the genetic diversity (29), spatial population structure (30), gene flow patterns (31, 32), incursion pathways (32–34), as well as cold adaptation (35, 36) of *Ae. albopictus*. However, limited numbers of mosquito populations have been sampled from the native population for studying the population genomics of this species, especially in China (34). Genome-wide SNPs could provide a valuable tool for identifying the genetic basis of important ecological adaptations, including traits related to invasion success and range expansion (37). Such information may provide the basis for novel vector-control strategies based on the genetic or chemical disruption of these adaptations.

This study was designed to investigate the genomic patterns of the spatial population genetic structure of *Ae. albopictus* across China. We used genome-wide SNPs to determine the distribution of genetic diversity and population structure of the *Ae. albopictus* populations across eight different geographical locations. Combined with the *cox*1 and microsatellite datasets from the same populations in our previous study (27), we compared the spatial genetic structure exhibited by the genome-wide SNPs, *cox*1, and microsatellite markers in the populations.

## Methods

### Mosquito sampling and DNA extraction

**Figure 1:**
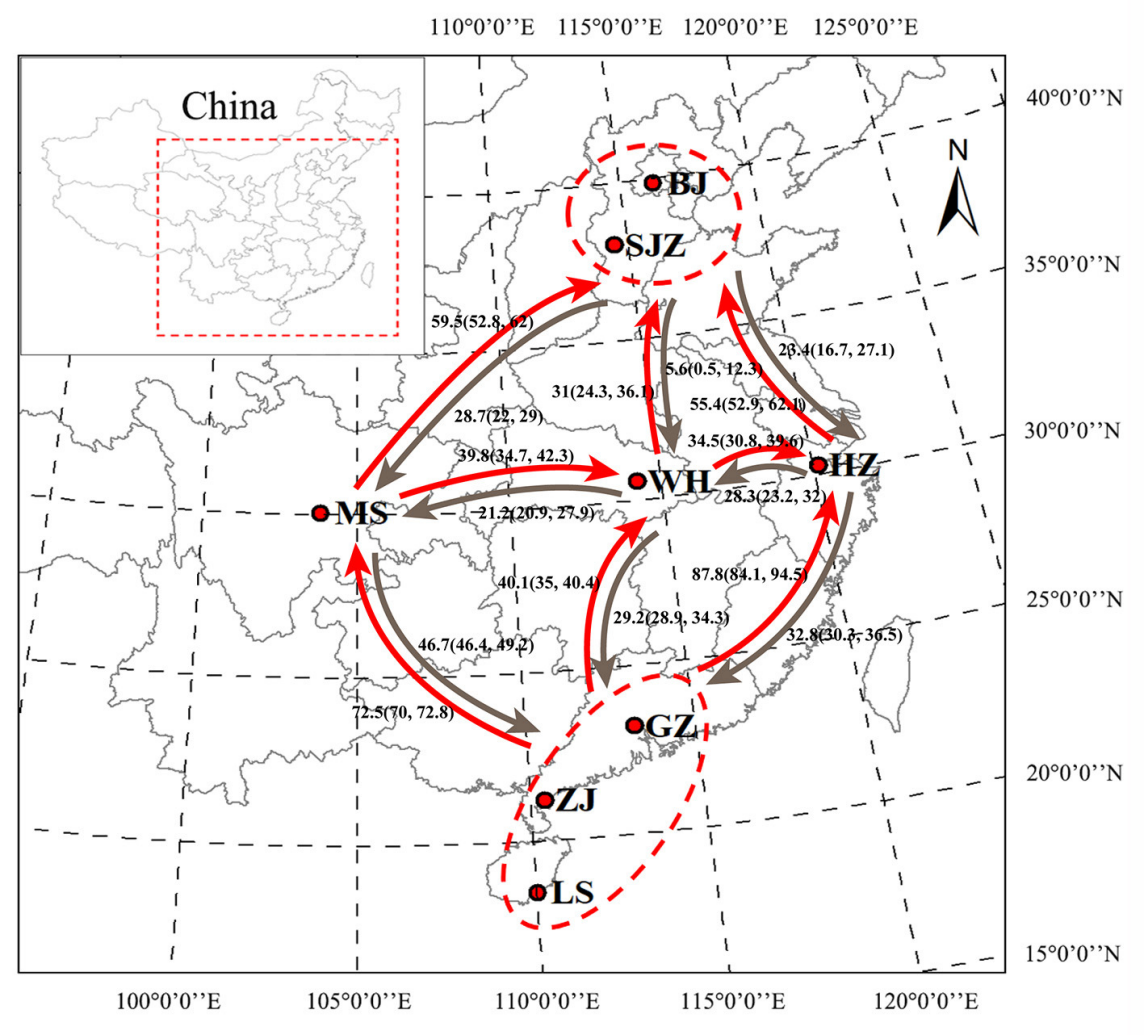
Geographical locations of *Ae. albopictus* sampling sites in China. Bayesian estimates of historical asymmetrical migration between five locality groups of *Ae. albopictus* on the basis of the genome-wide SNPs. The numbers indicate migration rate, and arrows indicate the direction of migration. BJ, Beijing; SJZ, Shijiazhuang; HZ, Hangzhou; WH, Wuhan; MS, Meishan; GZ, Guangzhou; ZJ, Zhanjiang; LS, Lingshui. The map was generated using ArcMap 10.2.2 software. Map source: ESRI (available at: www.esri.com).

Between August 2016 and September 2018, we collected *Ae. albopictus* adult female mosquitoes from different geographical clusters in eight locations (Beijing, BJ; Shijiazhuang, SJZ; Hangzhou, HZ; Wuhan, WH; Meishan, MS; Guangzhou, GZ; Zhanjiang, ZJ; and Lingshui, LS) from our previous study (27) in China (Figure 1). At each location, 8–12 households or collection points 400–3,000 m apart were selected randomly for obtaining adult mosquitoes, and 20–32 *Ae. albopictus* female mosquitoes from each location, with 2–3 individuals per collection point, were used for DNA extraction and genetic analysis. All adult mosquito specimens were identified using morphology under a stereomicroscope (Nikon, Tokyo, Japan) and *cox*1 sequencing. Sampling bias was examined using previous microsatellite data (27). Total genomic DNA was individually extracted using the Insect DNA Kit (Omega Bio-tek, GA, USA), according to the manufacturer's instructions.

### PCR amplification and mtDNA sequencing

SNPs in the mitochondrial gene *cox*1 of the mosquito specimens were examined. PCR was performed to amplify a 796 bp fragment in the 5′ *cox*1 region of the mtDNA by using the DNA primer pairs 2027F (5′-CCCGTATTAGCCGGAGCTAT-3′) and 2886R (5′-ATGGGGAAAGAAGGAGTTCG-3′) (16). The 25 μl reaction mixture contained 40 ng of genomic DNA, 12.5 μl of 2 × PCR Master Mix (Promega, WI, USA), 1 μl each of the forward and reverse primers at 10 μmol/l, and nuclease-free water. The PCR conditions were as follows: 95°C for 5 min; 35 cycles of 95°C for 30 s, 60°C for 30 s, and 72°C for 1 min; and a final extension at 72°C for 10 min. The amplified fragments were run on a 1% agarose gel to check integrity, stained with ethidium bromide, and analyzed under UV light. The PCR products were purified using a gel extraction kit (Omega Bio-tek, GA, USA) and sequenced with PCR primer 2027F by using the ABI 3730XL automatic sequencer (Applied Biosystems, CA, USA).

### Library construction for RAD-seq

Samples with total DNA quantity >1 μg were sequenced using RAD-seq, and the sample sizes from the eight locations for library construction were 13 (BJ), 13 (SJZ), 13 (HZ), 12 (WH), 13 (MS), 14 (GZ), 15 (ZJ), and 12 (LS). One microgram of genomic DNA was digested with *Eco*RI (New England Biolabs, MA, USA), which identifies the 5′-GAATTC-3′ sequence. The Illumina P1 adapter (BGI, Shenzhen, China) was ligated to the digested DNA. Then, the products from different samples were pooled and randomly fragmented using Covaris E210 (Covaris, MA, USA) with agarose gel selection for 300–500 bp. The products were purified using the QIAquick PCR Purification Kit (Qiagen, CA, USA). The fragments were end-repaired using End Repair Mix (Qiagen) and then purified. The repaired DNA was combined with A-Tailing Mix (Qiagen); Illumina P2 adaptors (BGI) were ligated to the adenylated 3′ ends of the DNA, followed by product purification. PCR amplification with PCR Primer Cocktail and PCR Master Mix (NEB, MA, USA) was performed to enrich the adapter-ligated DNA fragments. The PCR conditions were as follows: 98°C for 30 s; 15 cycles of 98°C for 10 s, 65°C for 30 s, and 72°C for 30 s; and a final extension at 72°C for 5 min. The PCR products were selected using agarose gel electrophoresis with target fragments and then purified. The library was quantified using the Agilent Technologies 2100 bioanalyzer (Agilent Technologies, CA, USA) and ABI StepOnePlus Real-Time PCR System (Applied Biosystems). The libraries, with 16 samples pooled per library, were pair-end sequenced using the PE150 strategy on the HiSeq X Ten platform (Illumina, CA, USA).

### Sequence alignment and SNP identification

The filtered reads from all mosquito specimens were aligned to the reference genome of *Aedes albopictus* Foshan strain (GenBank accession: GCA_001444175.2) (38) and C6/36 cell line (GenBank accession: GCA_001876365.2) (39) with the Burrows–Wheeler Aligner bwa-0.7.17 software (bwa mem –M -t 5 -T 20) (40). The aligned.bam files were sorted and indexed using SAMtools v1.9 (41). GATK modules RealignerTargetCreator, IndelRealigner, BaseRecalibrator, and ApplyBQSR were used to process the.bam files (42). Then, HaplotypeCaller of GATK was used for variant calling of each sample (42). In this study, only SNPs were detected, and other complex events such as indels and multi-nucleotide polymorphisms were ignored. For instance, loci with more than two alleles were discarded to avoid potential sequencing errors. SNPs with global MAF > 0.05 across samples were retained to reduce false SNP identification (43). In addition, SNPs with a minimum genotyping rate of 80% within each population were retained. The following quality controls were also used for SNP calling: (a) mapping quality ≥55, (b) coverage depth >200 and <,000, and (c) Phred quality score for the assertion made in alternate bases >100. The SNPs were further filtered by removing the loci out of Hardy-Weinberg equilibrium with VCFtools v0.1.16 (44) and linkage disequilibrium with the parameter (–indep-pairwise 50 10 0.1) by using PLINK v1.9 (45) and then retained in the VCF output file. We only retained SNPs that were successfully genotyped in 50% of the individuals, a minimum quality score of 30, and a minimum read depth of 3 for further analysis.

### Data analysis

The *cox*1 sequences from 231 mosquitoes were aligned using Clustal W multiple alignment in BioEdit version 7.2.5 (46). The number of segregating sites (*S*), haplotype diversity (*Hd*), average number of nucleotide differences (*k*), and nucleotide diversity (π) within each population were determined using DnaSP v5.10.1 (47). To determine the genealogical relationships among the haplotypes, a haplotype network was constructed using a statistical parsimony algorithm implemented in TCS v1.21 (48). The minimum number of mutational steps between sequences was calculated with >95% confidence.

The filtered VCF file containing the genome-wide SNPs was analyzed for the population genetics of *Ae. albopictus*. The SNP dataset was analyzed as follows: PGDSPIDER v.2.0.5.0 (49) was used to reformat the VCF files into ARP format files for Arlequin. Deviations from selective neutrality were tested using Fu's *F*_s_ statistic (50) and Tajima's D (51). The neutrality test was performed for each population to examine population expansion. The Bayesian model-based clustering program STRUCTURE v2.3 and the Maximum-Likelihood (ML) clustering program ADMIXTURE v1.3.0 (52) were used to infer the cryptic genetic structure. On the basis of the genome-wide SNPs, *cox*1 data, and microsatellite data from our previous study, Bayesian clustering analysis was performed by conducting 20 independent runs for each K = 1 to 8, using a “burn-in” value of 50,000 iterations followed by 200,000 repetitions with STRUCTURE v2.3 (53). The optimal number of clusters (K) was determined using the Delta K method of Evanno et al. (54). The results of the clustering analysis were visualized using the ggplot2 package in R v4.0.3 (55). VCFtools v0.1.16 (44) was used to reformat the VCF file of the SNP data into PLINK format files (MAP/PED) for ADMIXTURE. Furthermore, average pairwise *F*_ST_ was calculated between each pair of sampling populations, and the Mantel test and AMOVA were conducted using Arlequin v3.5.2.2 (56). The gene flow and migration rates between all pairwise populations were estimated using the Bayesian coalescence-based approach implemented in LAMARC v2.1.10 (57). The Adegenet package (58) in R software (55) was used to perform principal component analysis (PCA) and discriminant analysis of principal components (DAPC). Neighbor-joining (NJ) and ML phylogenetic trees were constructed using MEGA 7.0 (59), according to the Kimura 2-parameter (K2P) model (bootstrap = 100) for *cox*1, microsatellite, and genome-wide SNP data. The best-fitting model for evolution and model parameters was determined using the Bayesian Information Criterion in jModelTest 2.1.10 (60). A significance level at *P* < 0.05 was set for all statistical tests, and sequential Bonferroni correction (61) was used when significant correlations were detected between the paired data.

## Results

### Genetic variation and haplotype network based on *Cox*1

PCR amplification and sequencing of the mitochondrial *cox*1 gene resulted in the detection of a 765 bp fragment in each specimen, with no insertions or deletions. All 231 sequences were identical or possessed >99% similarity to *Ae. albopictus* (GenBank: KR068634). According to *cox*1 sequencing and identification of cryptic species in our previous study (27), no cryptic *Ae. albopictus* species were found in all populations. Twenty-seven variable sites were detected, and 20 of them were parsimony-informative. The ZJ population had the highest number of polymorphic sites (*S* = 10, the same as the GZ population), haplotype diversity (*H*_d_ = 0.890), and nucleotide diversity (π = 0.248) and the highest average number of nucleotide differences (*k* = 1.897). However, the BJ population had the lowest values for these genetic indices, as it had only two haplotypes (Table 1).

**Table 1:** Population information and genetic polymorphism based on *cox*1 of *Ae. albopictus* in China.

**Populations**	**Abbreviation**	**Latitude**	**longitude**	**n**	***S***	**h**	***H*** **_d_**	***k***	**π**
Beijing	BJ	39°51′36^′′^N	116°11′45^′′^E	21	1	2	0.095	0.095	0.012
Shijiazhuang	SJZ	37°54′55^′′^N	114°27′49”E	32	5	5	0.532	1.399	0.183
Hangzhou	HZ	30°18′42^′′^N	120°07′09^′′^E	25	5	6	0.533	0.613	0.080
Wuhan	WH	30°30′30^′′^N	114°22′39^′′^E	30	4	5	0.453	0.501	0.066
Meishan	MS	30°11′55^′′^N	103°52′01^′′^E	30	6	6	0.809	1.554	0.203
Guangzhou	GZ	23°11′15^′′^N	113°19′42^′′^E	32	10	10	0.742	1.151	0.150
Zhanjiang	ZJ	21°05′37^′′^N	109°42′60^′′^E	30	10	10	0.890	1.897	0.248
Lingshui	LS	18°30′27^′′^N	110°01′59^′′^E	31	8	8	0.811	1.729	0.226

n, number of samples; S, number of segregating sites; h, number of haplotypes; H_d_, haplotype diversity; k, average number of nucleotide differences; π, nucleotide diversity (× 10^2^, average number of nucleotide differences per site).

A total of 32 haplotypes of *cox*1 mtDNA were detected in the 231 specimens (GenBank: MT188111–MT188130, MT755918–MT755929). To determine the relationships between the specimens or haplotypes, we constructed a median-joining network using the haplotypes on the basis of sequence variation (Supplementary Figure S1). Most of the specimens were identified as H01 (45.9%) in the *Ae. albopictus* populations, but H01 was not detected in the LS population (Supplementary Figure S1). Some haplotypes were unique to a specific population (for example, H12, H13, and H15 in MS; H16, H17, H18, H19, H20, and H21 in GZ; H22, H23, H24, H26, and H27 in ZJ; and H28, H29, H30, H31, and H32 in LS), whereas the H05 and H06 haplotypes appeared simultaneously in specimens of the SJZ, ZJ, and LS populations (Supplementary Figure S1).

### RAD-seq and data filtering

The average number of filtered quality reads per individual was approximately 24.79 million reads (range: 4,630,354–42,550,002; Supplementary Table S1). The Q20_rate (ratio of bases with a quality value >20 in all reads to the total length of reads) was more than 96%. Quality filtering yielded 33,769 SNPs, and a total of 9,968 SNPs were retained after checking for Hardy-Weinberg equilibrium and linkage disequilibrium. The mean and median values of SNP coverage per sample were shown using box-and-whisker plots (Supplementary Figure S2). Four samples had <50% of the loci genotyped, and 865 SNPs identified in <50% samples were removed; the remaining 9103 SNPs from the 101 samples were used for further population genetics analysis. A summary of the statistics for the counts of putative SNP loci and final counts of candidate SNPs after each filtering step is available in Table 2.

**Table 2:** Summary statistics for counts of putative SNP loci and final counts of candidate SNPs after different filtering steps.

**Filtering step**	**SNP counts**
Biallelic variants	60,982,824
Global minor allele frequency (MAF > 0.05)	38,300,712
Coverage ratio of samples for each pop ≥ 80%	156,516
Mapping quality ≥ 55; 200 < Coverage depth < 40,000;	33,769
Phred-scaled quality score for the assertion made in alternate bases > 100	
Reservation of Hardy-Weinberg equilibrium loci	24,136
Removal of linkage disequilibrium loci	9,968
Removal Loci genotyped < 50%, reads depth < 3	9,103

### Neutrality test, gene flow, and population genetic analysis

The neutrality of the SNP dataset was checked using Tajima's *D* and Fu's *F*_s_ tests. Tajima's *D* tests for all populations were not statistically significant (Table 3); this indicates that the populations are in genetic equilibrium, which is consistent with the neutral mutation hypothesis. Likewise, Fu's *F*_s_ tests were not statistically significant (Table 3) and rejected the population expansion/bottleneck model. Tajima's *D* tests and Fu's *F*_s_ tests of the *cox*1 data for the HZ and GZ populations were statistically significant (Table 3).

**Table 3:** Neutrality tests based on genome-wide SNPs and *cox*1 of *Ae. albopictus* populations in China.

**Populations**	**Genome-wide SNPs**		***cox*** **1**	
	**Tajima's D**	**Fu's *F*_s_**	**Tajima's *D***	**Fu's *F*_s_**
BJ	−0.682	1.711	−1.164	−0.919
SJZ	−0.675	1.311	0.341	0.347
HZ	−0.684	1.604	−1.538^*^	−3.491^*^
WH	−0.796	1.694	−1.293	−2.493^*^
MS	−0.688	1.523	0.074	−0.353
GZ	−0.773	1.245	−1.675^*^	−5.849^*^
ZJ	−0.806	0.298	−0.788	−3.406^*^
LS	−0.512	1.710	−0.412	−1.734

* *P* < 0.05.

On the basis of the SNP dataset, all 28 pairwise tests of genetic differentiation were significant at *P* < 0.05 after Bonferroni correction, and the pairwise *F*_ST_ values ranged from 0.020 (between WH and GZ) to 0.106 (between HZ and ZJ); a few pairwise tests showed no significance on the basis of the *cox*1 and microsatellite data (Supplementary Table S2). The Mantel test showed a statistically significant correlation (SNPs: y = 0.0177x−0.0618, *R*^2^ = 0.190, *P* = 0.006; microsatellites: y = 0.008x−0.0341, *R*^2^ = 0.288, *P* = 0.002) between the genetic distance [y, estimated as *F*_ST_/(1 – *F*_ST_)] and the geographical distance [x, estimated as Ln (km)] of populations on the basis of the SNP and microsatellite data, with no significant correlation (y = 0.1185x−0.5812, *R*^2^ = 0.117, *P* = 0.054) on the basis of the *cox*1 data (Supplementary Figure S3). The LAMARC analysis showed that the historical gene flow rates ranged from 5.6 to 87.8. An asymmetric gene flow was detected among populations, and it was higher from south to north and from west to east regions than the opposite directions (Figure 1).

**Figure 2:**
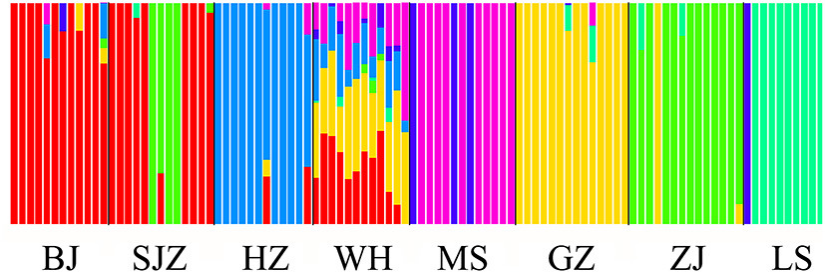
Structure analysis of the SNP dataset on the basis of the Maximum-Likelihood clustering method.

**Figure 3:**
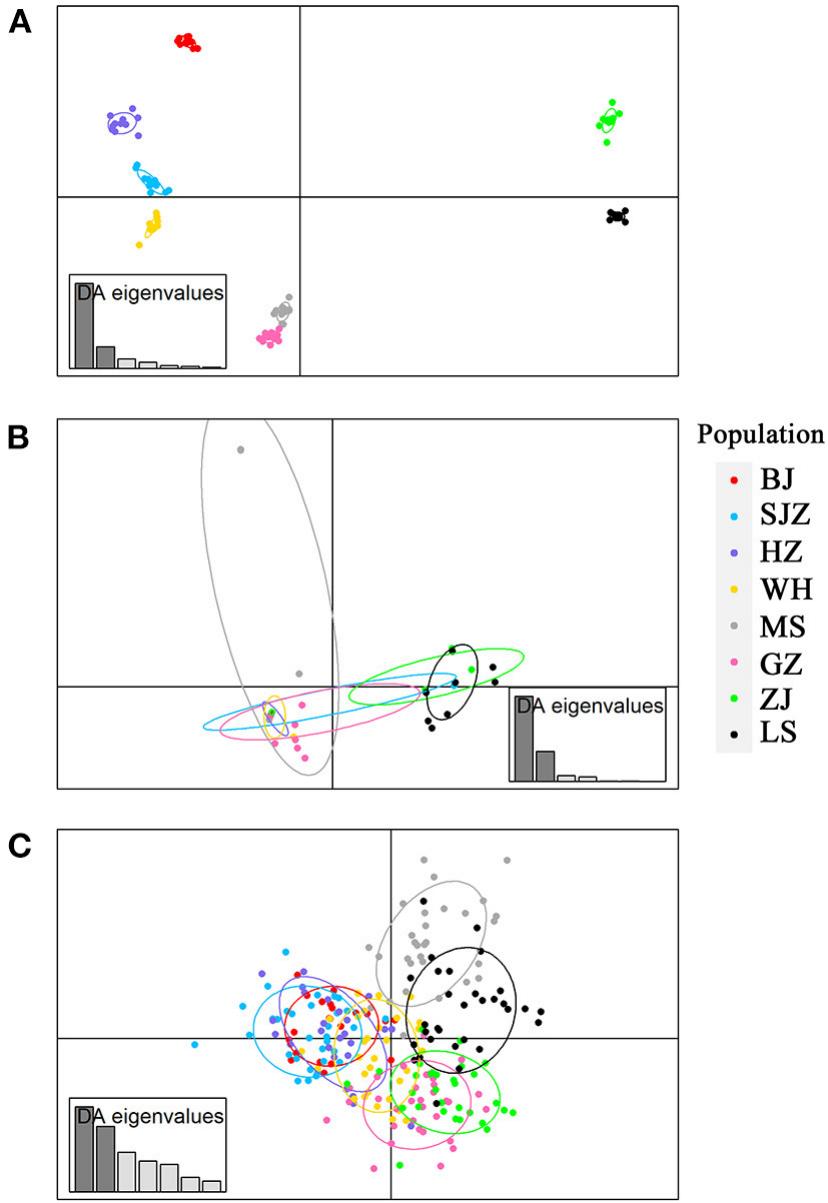
Discriminant analysis of principal components (DAPC) plots for genome-wide SNPs **(A)**, *cox*1 **(B)** and microsatellites **(C)** in eight field populations of *Ae. albopictus* in China. The plots show the relationship between individuals belonging to eight different populations (represented by colored dots, where the color of a dot corresponds to a population).

**Figure 4:**
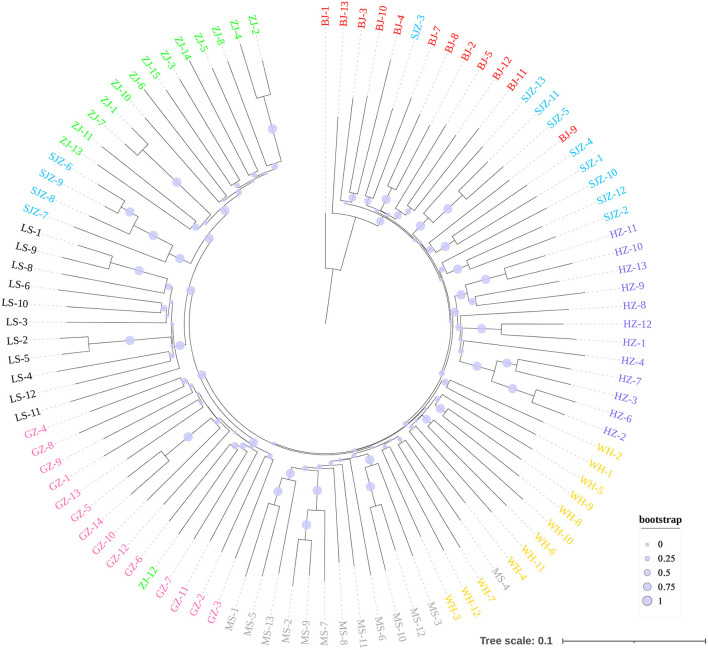
Neighbor-Joining phylogenetic tree constructed using 9,103 genome-wide SNPs.

**Figure 5:**
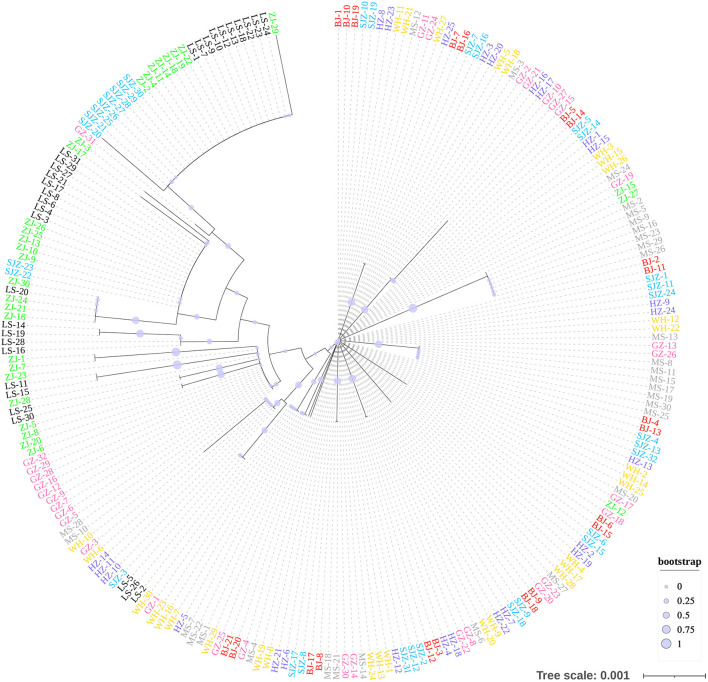
Neighbor-Joining phylogenetic tree constructed using the *cox*1 data.

**Figure 6:**
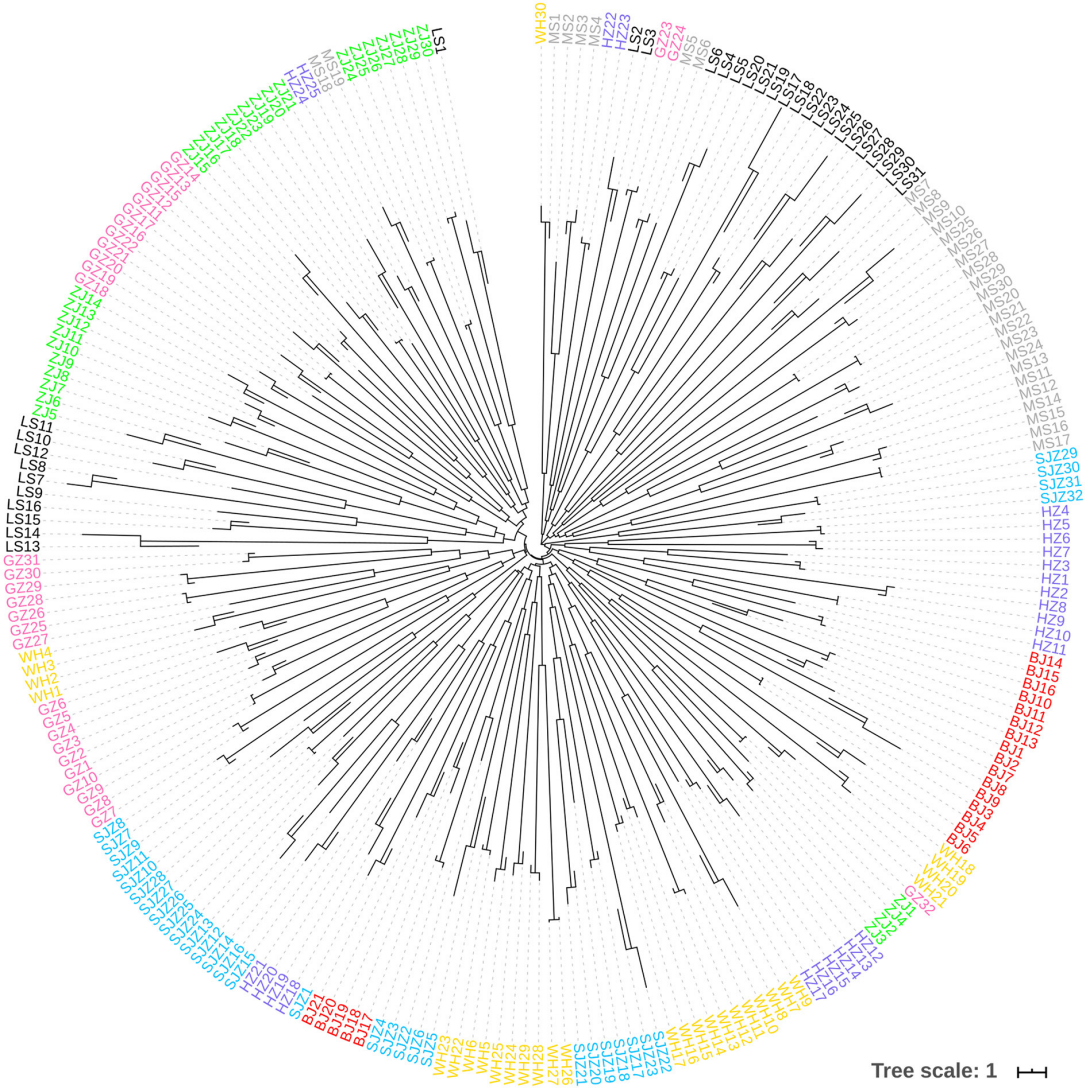
Neighbor-Joining phylogenetic tree constructed using the microsatellite data.

The Bayesian clustering analysis based on the datasets of the three DNA markers showed that the ZJ and LS samples were clustered together and separated from the other populations when K = 2. In addition, the optimal cluster numbers (K) determined using the Delta K method for SNPs, *cox*1, and microsatellite data were 2, 2, and 3, respectively (Supplementary Figure S4). The ML clustering analysis based on the SNP dataset showed obvious division among the samples when K = 7, as the cross-validation error was the lowest (Figure 2). All samples were clearly separated on the basis of the SNP data rather than the *cox*1 and microsatellite data in the coordinate system of the DAPC (Figure 3). A large number of dots representing the samples of the DAPC based on the *cox*1 data (Figure 3B) overlapped because of many identical haplotypes in each population. The division of samples by DAPC on the basis of the microsatellite data was unclear (Figure 3C). The PCA results via the two-dimensional plots (Supplementary Figure S5) were consistent with those of the DAPC. The NJ phylogenetic tree (Figure 4) and ML phylogenetic tree (Supplementary Figure S6) constructed using the SNP dataset showed that each sample could be clearly separated and classified from its original population. In this respect, the SNPs outperformed *cox*1 (Figure 5; Supplementary Figure S7) and microsatellites (Figure 6). The phylogenetic analysis based on the SNP dataset showed that some samples from the SJZ had a close relationship with those from LS and ZJ, which is congruent with the H05 and H06 haplotypes limited to these populations in the *cox*1 haplotype network. The AMOVA results (Table 4) indicated that most variations in *Ae. albopictus* were within populations: 94.08%, SNPs; 67.24%, *cox*1; and 97.82%, microsatellites; variations among the populations accounted for 5.92% (genome-wide SNPs), 32.76% (*cox*1), and 2.18% (microsatellites) of the total variation. The fixation index among the populations showed significant genetic variation on the basis of Fisher's exact test for the three types of DNA marker data.

**Table 4:** Analysis of molecular variance (AMOVA) based on the three DNA markers of *Ae. albopictus* populations in China.

**DNA marker**	**Source of variation**	***df***	**SS**	**Variance components**	**Percentage of variation**	**Fixation index**	***P*** **-value**
Genome-wide SNPs	Among populations	7	676.780	3.390	5.92	0.059	*P* < 0.001
	Within populations	93	5,014.240	53.917	94.08		
	Total	100	5,691.020	57.307			
*cox*1	Among populations	7	61.860	0.286	32.76	0.328	*P* < 0.001
	Within populations	223	131.079	0.588	67.24		
	Total	230	192.939	0.874			
Microsatellites	Among populations	7	72.094	0.101	2.18	0.022	*P* < 0.001
	Within populations	223	2,046.194	4.507	97.82		
	Total	230	2,118.288	4.608			

df, degrees of freedom; SS, sum of square.

## Discussion

Global warming and rapid development of transportation have caused the distribution of *Ae. albopictus* to continuously expand to the north of China (11). The invasion of *Ae. albopictus* has increased the potential risks of mosquito-borne diseases in some non-endemic areas in China (62). High-throughput genotyping with next-generation sequencing technology is being increasingly used to study the population genetics of disease vectors (31, 63, 64). An appropriate genotyping strategy and fine-population genetic analysis of *Ae. albopictus* would help us to understand the dynamics of its population spread and establishment and design strategies for controlling the vectors and mosquito-borne diseases (5, 22, 65).

In this study, the dominate haplotype H01 of *cox*1 was detected in seven geographical *Ae. albopictus* populations in mainland China, suggesting H01 is relatively conservative, as observed in another study of the *Ae. albopictus* population in China (66). The haplotype diversity is richer in southern China, which is similar to the findings of our previous study and other studies (14, 16, 27). One possible explanation is that the environment and climate in southern China are more conducive to survival, reproduction, and continuous dispersion, whereas the relatively dry and cold climate in northern China could result in lower allele richness and genetic diversity in *Ae. albopictus* populations (14, 16).

The results of Tajima's *D* and Fu's *F*_s_ tests based on the genome-wide SNPs indicated demographic equilibrium and no population bottleneck/expansion. Thus, these populations may exist stably and not have invaded recently as new founding populations (18). Previous studies have indicated two gene pools on the basis of microsatellites from the *Ae. albopictus* population in China (14, 27). The genome-wide SNPs results divided the eight Chinese *Ae. albopictus* populations into seven genetic clusters, indicating the presence of seven gene pools in the sampled areas. Individuals sampled from six populations were assigned to six different clusters, corresponding to actual geographical locations; in contrast, the WH and SJZ populations were mixed with individuals from other gene pools, indicating the coexistence of different genetic units in these locations, similar to other vector species in China (67).

Our results showed that the *F*_ST_-values were low but significantly different among all populations, and some pairs were not significantly different in our previous study using microsatellites, for example, BJ and SJZ, HZ and SJZ, and GZ and ZJ populations. Compared to the results of clustering and population differentiation on the basis of *cox*1 and microsatellites, the genome-wide SNPs exhibited stronger separation between populations than the other markers, which is consistent with the results of previous studies (64, 68, 69). On the basis of the SNPs, the LS and ZJ populations were more genetically different than the other populations. Several factors may contribute to the genetic differentiation and shape the structure of the two southern populations and other populations in China: (1) The tropical climate characteristics (hot and humid) of Hainan Island and southern China facilitate the breeding and development of *Ae. albopictus* (70–73). (2) The thriving border trades and many famous tourist attractions in southern China provide many pathways for the introduction and movement of this species from other Southeast Asian countries, thus increasing genetic diversity (74). (3) The geographical isolation of Hainan Island is probably an important factor that limits species dispersal to other places, thus affecting the gene flow and distribution of genetic diversity (66, 75).

The population analysis using the SNP dataset showed that the SJZ population specimens had close evolutionary relationships with the ZJ and LS population specimens, which also confirms that they have the same specific *cox*1 haplotypes (H05 and H06). We speculated that several mosquitoes from individual SJZ collection points may have originated from ZJ and LS populations because of human-assisted transportation over long distances. When compared with the customs of provincial capital cities such as BJ, HZ, and WH, the customs of SJZ may have a relatively loose control on commercial transportation and species invasion. The population structure bar plots generated using STRUCTURE on the basis of the SNPs showed that the WH population had the highest shared SNP alleles with neighboring populations; this is possibly because Wuhan is a central city in China and a transportation hub, leading to an increase in mosquito migration and gene flow. The overall gene flow showed a trend from south to north and from west to east, which is consistent with the trend determined using a large number of samples in our previous study (27). Similar dispersal behavior can produce similar patterns of differentiation (76). Mosquitoes spread from south to north through active dispersal affected by global warming and passive dispersal caused by human activities (77–80). This also explains the reports of some dengue cases in new non-endemic areas in recent years (62, 81).

Genome-wide SNPs identified with next-generation sequencing (such as RAD-seq) that are used to analyze population genetics have two major advantages when compared with microsatellites and *cox*1: the need for smaller sample sizes and no need for prior genomic information (82, 83). SNP analyses have corroborated microsatellite-based findings and provided a more accurate and robust population structure than microsatellite analyses (68, 84). In our previous study (27), a set of polymorphic microsatellites were an economical choice for a large-scale population genetic clustering analysis, but not suitable for finer genetic structural analysis of relatively few populations of *Ae. albopictus*. It is important to consider a very high probability of incorrect conclusions from *cox*1 data when the data alone are used to infer the population history of arthropods, due to indirect selection on mtDNA *cox*1 arising from linkage disequilibrium with inherited microorganisms (such as *Wolbachia*) in *Ae. albopictus* (24). The Mantel test detected significant positive relationships between genetic and geographical distance, whereas no such relationships were evident from the *cox*1 data. Therefore, *cox*1 data could be problematic when performing the Mantel test for isolation by distance (IBD), as they often do not provide reliable information on the true dispersal potential of a species (25).

Currently, next-generation sequencing costs have decreased dramatically, and several reference genome assemblies of *Ae. albopictus* are available, including reference genomes AaloF1 (38) and AalbF2 (85) assembled from the Chinese Foshan strain and MNAF02 assembled from the C6/36 cell line (39); these assemblies greatly facilitate allele-specific measurements (65). In this study, we used two assembled genomes as references, and several samples were aligned with only 50% or less reads to the reference genome. This suggests reference bias, although the reference genome AaloF1 is based on a laboratory strain from a local population in China. Indeed, a previous study also obtained a low read mapping rate (25%) based on the primary reference assembly (29). However, the ~9,000 high-quality SNPs obtained in the study should be enough to assess the genetic diversity and population structure of *Ae. albopictus*. Overall, the genomic patterns identified in this study can help to identify biosecurity threats in *Ae. albopictus* by revealing a likely degree of gene flow (31, 80). The fine spatial genetic structure and gene flow data based on the genome-wide SNPs, in combination with other related factors such as mosquito density, climate, and rainfall, will be valuable for vector surveillance as well as epidemiological prediction and modeling of the incidence and spread of vector-borne diseases.

## Conclusions

Next-generation sequencing techniques such as RAD-seq provide an increasingly affordable approach for generating numerous genetic markers to study disease vector populations. A total of 9,103 SNP loci and seven gene pools were identified from *Ae. albopictus* specimens by using RAD-seq, and the SNP dataset showed shallow but significant genetic differentiation among the populations, except specimens from WH and SJZ. The LS and ZJ populations were isolated from the other populations and exhibited the effects of geographical distance and barriers to gene flow. The Mantel test indicated a positive correlation between genetic distance and geographical distance. However, asymmetric gene flow was detected among the populations, and it was higher from south to north and west to east regions than the opposite directions. The resolution of the population genetic structure inferred from the genome-wide SNPs was better than that from the *cox*1 data and a set of polymorphic microsatellites. The RAD-seq based approach demonstrates great potential for obtaining information on the spatial dynamics of population spread and establishment and designing new strategies for the control of vectors and mosquito-borne diseases.

## Data availability statement

The datasets presented in this study can be found in online repositories. The names of the repository/repositories and accession number(s) can be found in the article/Supplementary material.

## Author contributions

YW and XZ conceived and designed the experiments. YW, SH, JW, PF, YH, KH, and YC performed the experiments. YW and DZ analyzed the data. YW, XZ, DZ, and GZ wrote and revised the manuscript. All authors read and approved the final manuscript.

## Funding

This work was supported by the National Natural Science Foundation of China (No. 31630011), Natural Science Foundation of Guangdong Province (No. 2017A030313625), and the Science and Technology Planning Project of Guangzhou (No. 201804020084).

## Conflict of interest

The authors declare that the research was conducted in the absence of any commercial or financial relationships that could be construed as a potential conflict of interest.

## Publisher's note

All claims expressed in this article are solely those of the authors and do not necessarily represent those of their affiliated organizations, or those of the publisher, the editors and the reviewers. Any product that may be evaluated in this article, or claim that may be made by its manufacturer, is not guaranteed or endorsed by the publisher.
