# Supplementary figures and images for: Genome-wide SNPs reveal novel patterns of spatial genetic structure in Aedes albopictus (Diptera Culicidae) population in China
Source: Front Public Health. 2022 Nov 10;10:1028026. doi: 10.3389/fpubh.2022.1028026 (PMC9685676; doi:10.3389/fpubh.2022.1028026)

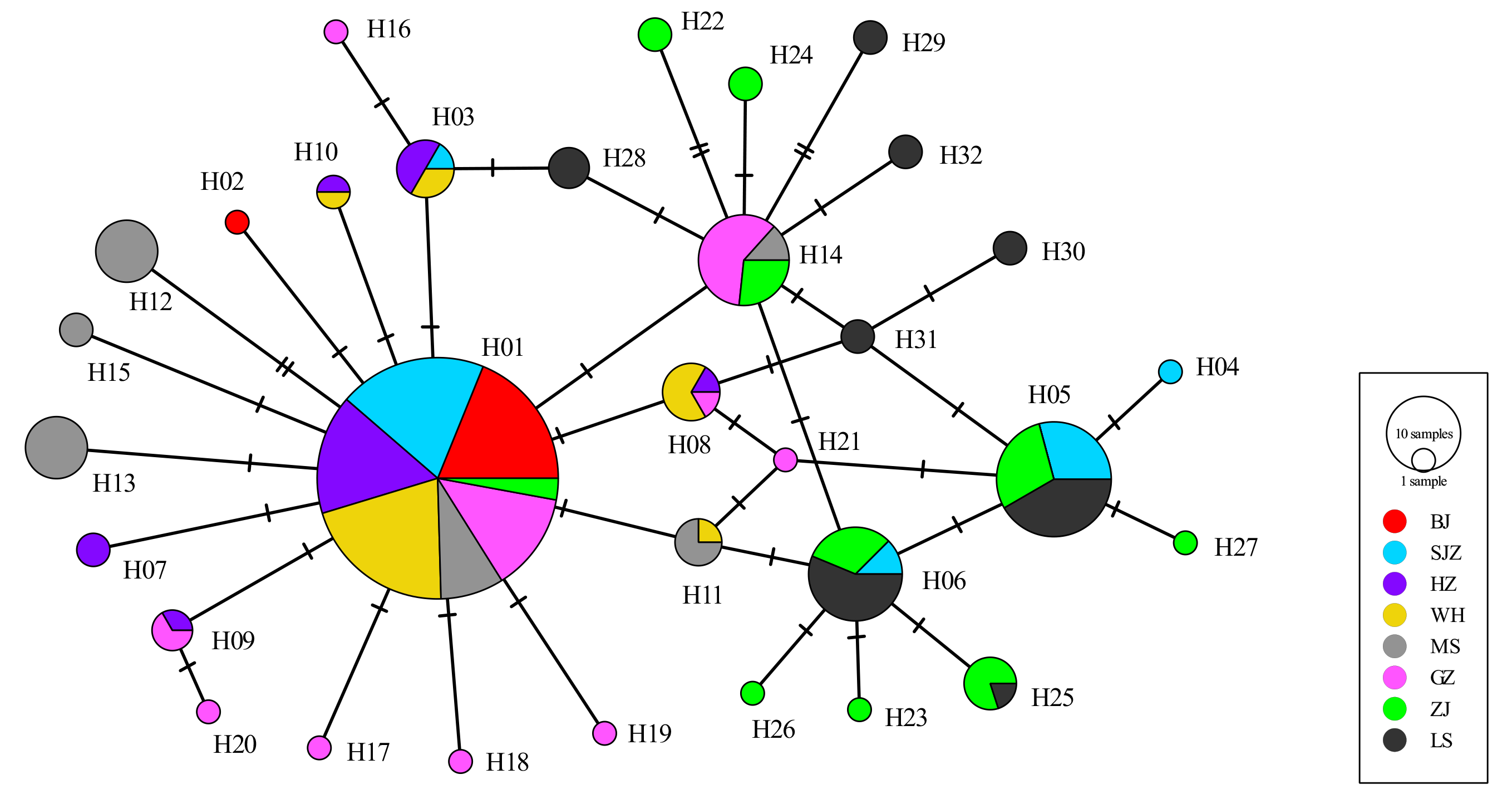

Supplement: Supplementary file 3 [file Image_1.TIF]

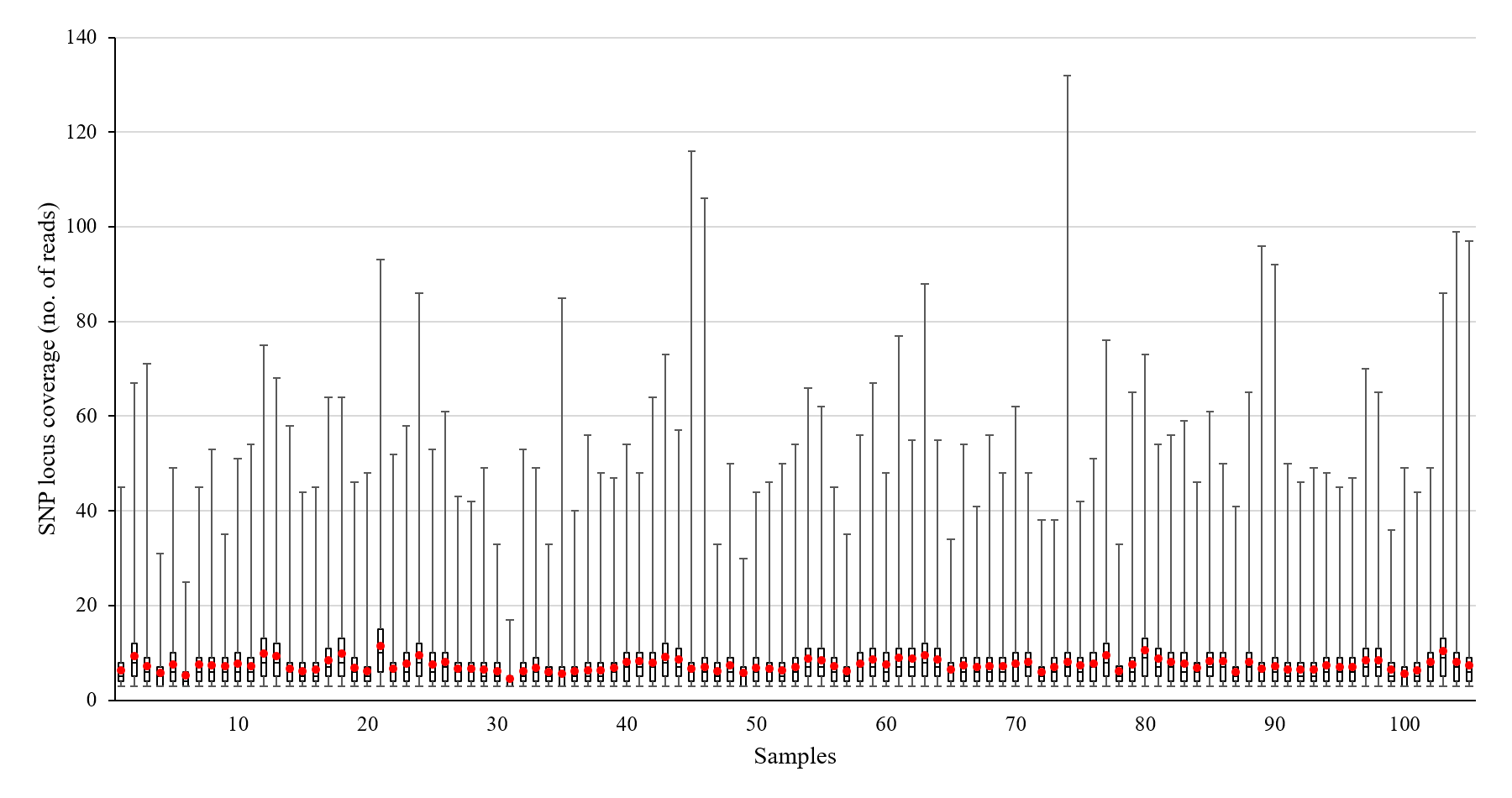

Supplement: Supplementary file 4 [file Image_2.TIF]

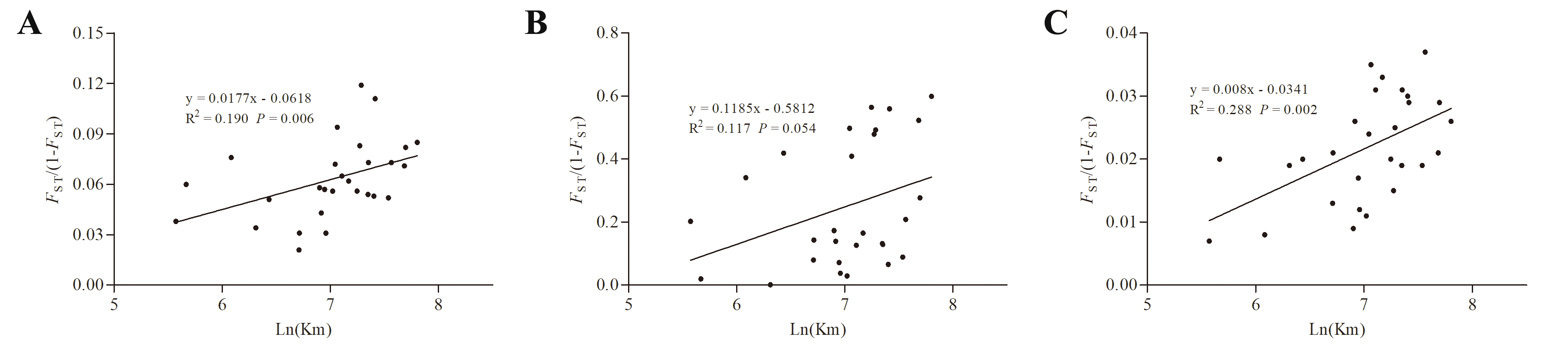

Supplement: Supplementary file 5 [file Image_3.TIF]

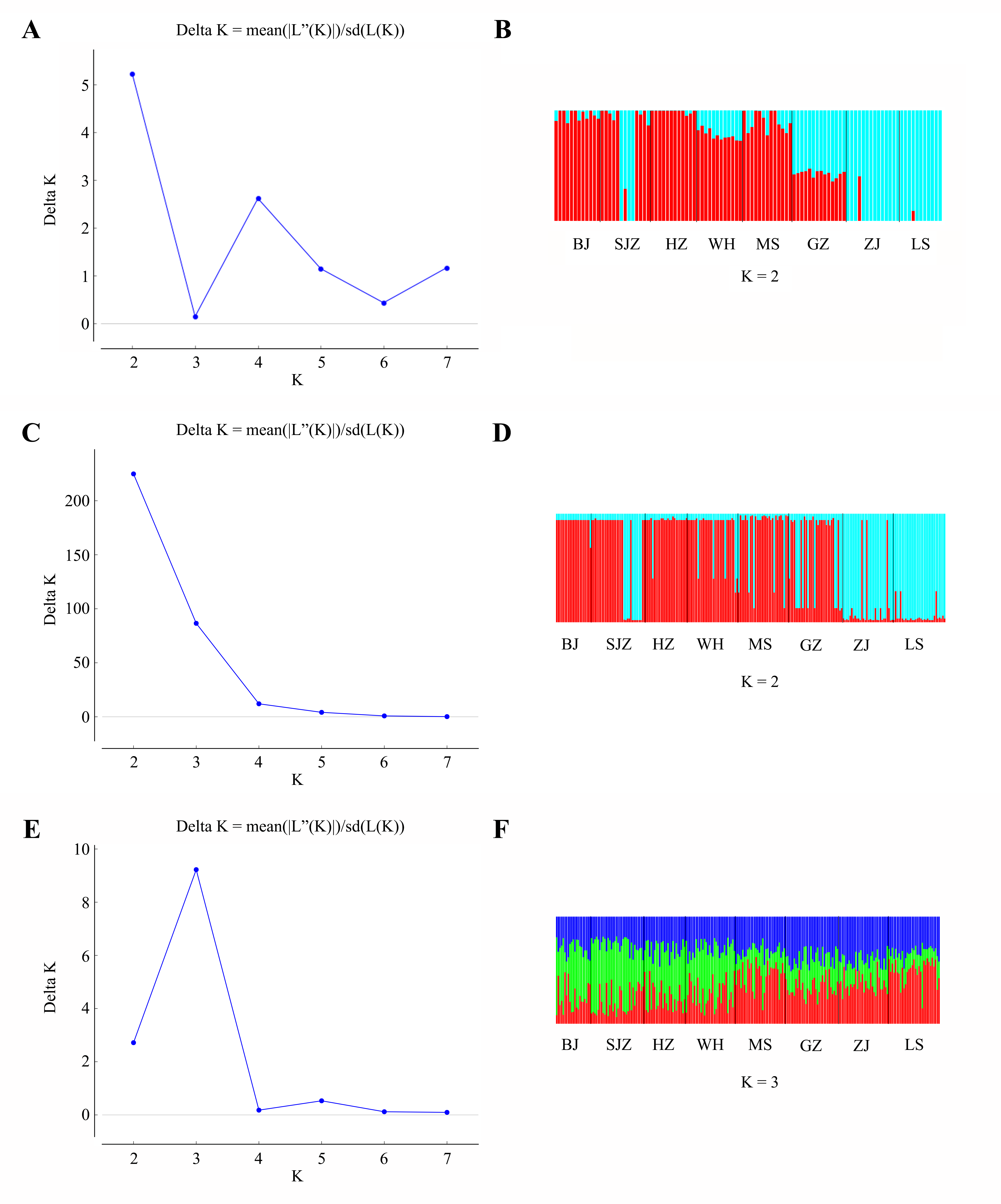

Supplement: Supplementary file 6 [file Image_4.TIF]

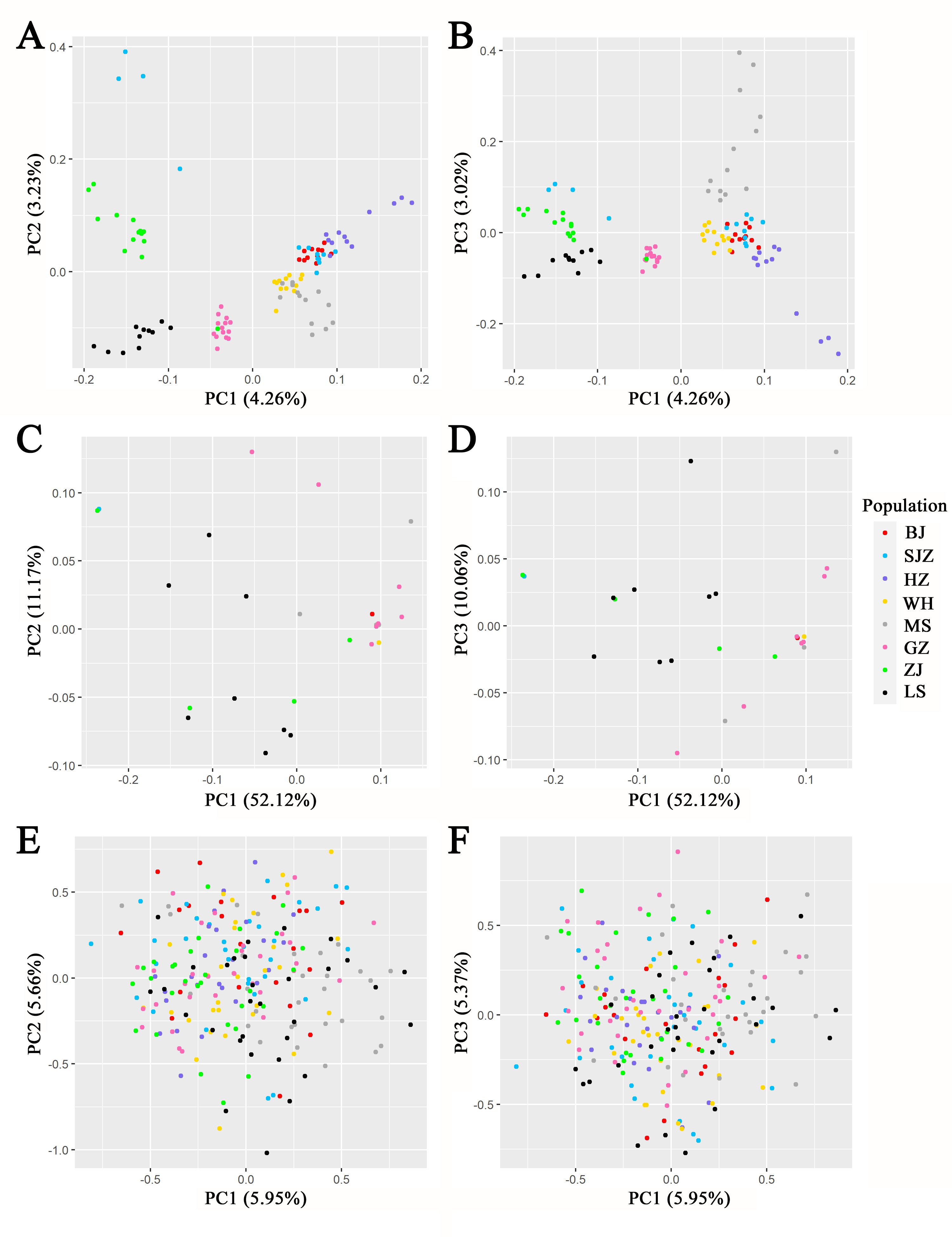

Supplement: Supplementary file 7 [file Image_5.TIF]

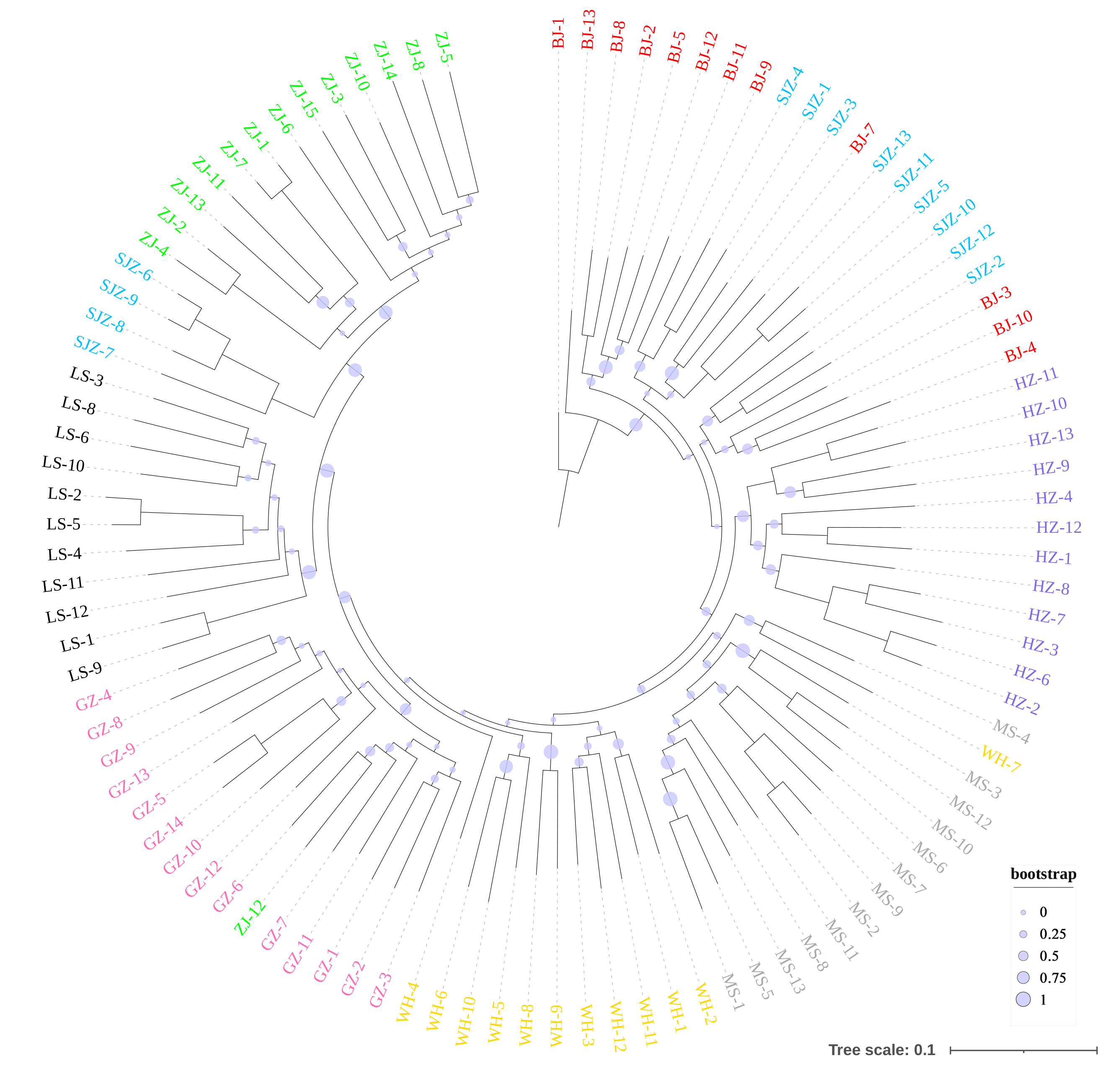

Supplement: Supplementary file 8 [file Image_6.TIF]

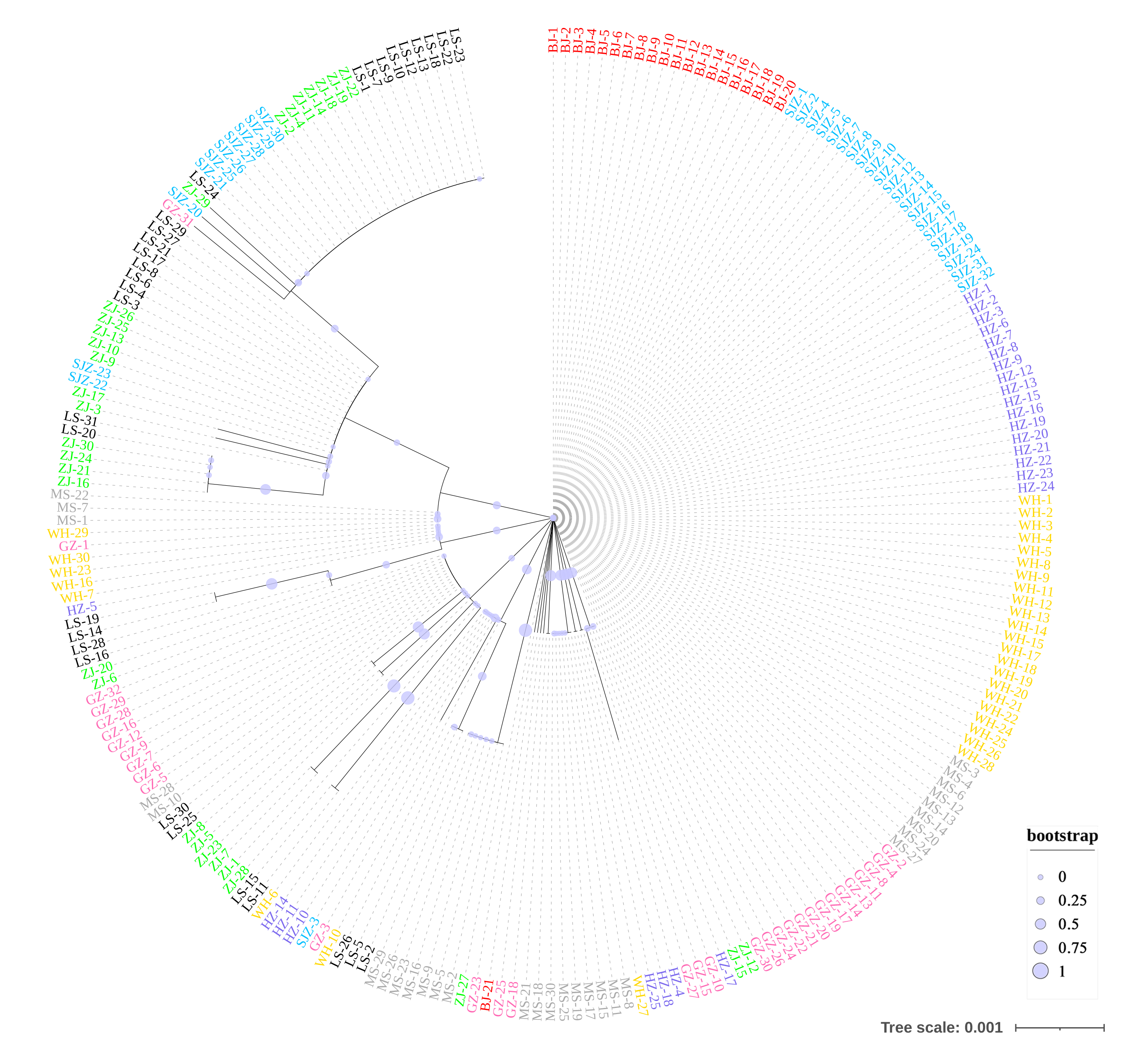

Supplement: Supplementary file 9 [file Image_7.TIF]
